# Postprandial glucose response to a low-sucrose chocolate spread in adults with type 1 diabetes: a randomized, double-blinded, cross-over, controlled trial

**DOI:** 10.1038/s41387-026-00429-7

**Published:** 2026-05-20

**Authors:** Naama Fisch-Shvalb, Revital Nimri, Moshe Phillip, Michal Yackobovitch-Gavan

**Affiliations:** 1 Institute of Endocrinology and Diabetes, Schneider Children’s Medical Center, Petach Tikva, Israel; 2 Department of Pediatrics, Gray Faculty of Medical & Health Sciences, Tel Aviv University, Tel Aviv, Israel; 3 Epidemiology and Preventive Medicine, School of Public Health, Gray Faculty of Medicine, Tel Aviv University, Tel Aviv, Israel

**Keywords:** Type 1 diabetes, Randomized controlled trials, Nutrition

## Abstract

**Background:**

Novel advances in food technology enable the production of foods with significantly reduced sucrose without the use of additives. We tested the glycemic response of subjects with Type 1 Diabetes (T1D) to a novel sucrose-reduced chocolate spread, as well as its acceptance and palatability.

**Methods:**

A randomized, double-blind, crossover, active-controlled trial comparing the glycemic response to two test-meals: 20 grams of the sugar-reduced spread (1.6 grams sucrose), and 20 grams of control chocolate spread, (11 grams sucrose) in people with T1D.

**Results:**

Thirty adults (50% males), aged 18–28 years (mean 23.0 ± 3.0), mean HbA1C of 55 ± 9 mmol/mol (7.2 ± 0.9%) were recruited. Postprandial glucose (PPG) excursions were significantly lower following consumption of the study spread compared to the control spread: pre-meal to peak glucose difference (mean 25.8 ± 34.9 mg/dL lower, *P* < 0.001), postprandial CGM iAUC (mean 2271 ± 3789 min×mg/dL, *P* = 0.003). Median time in range was higher for the study versus control spread (100% and 64.6%, respectively, *P* = 0.030). In the sweetness scale questionnaire, the study spread rated higher than the control (“right degree of sweetness” 53.3% and 26.7% respectively, *P* < 0.001).

**Conclusions:**

This new technology for producing sugar-reduced food is able to reduce PPG with a limited effect on palatability.

## Introduction

Intensive glucose management aimed at achieving target blood glucose levels is crucial for people with Type 1 Diabetes (T1D) to prevent both acute and long-term complications [1]. Post-meal blood glucose (postprandial glycemia, PPG) plays a significant role in overall glycemic control and glucose variability (GV) in T1D [2, 3]. According to the American Diabetes Association (ADA), postprandial glucose (PPG) levels should ideally remain below 180 mg/dL at any time after eating [1]. A yet more stringent target closer to normal postprandial levels observed in individuals without diabetes (under 7.8 mmol/L or 140 mg/dL within two hours of a meal) may provide greater benefits in preventing long-term complications [4]. Despite advancements in diabetes technologies such as continuous glucose monitors, insulin pumps, and automated insulin delivery systems, maintaining an optimal PPG profile remains challenging and significantly impacts glycemic variability [5, 6]. This variability contributes to vascular damage, raises the risk of hypoglycemia, and is associated with higher rates of mortality, cardiovascular disease, and cognitive decline [7].

Postprandial glucose excursions in T1D happen mainly due to a delay in subcutaneous insulin absorption and action, but also among other factors, depend on the meal composition. The glycemic index (GI) ranks foods based on acute glycemic response over a 2 h period of 50 g of available carbohydrates (CHO) of a test food compared with the reference standard glucose [8]. Glycemic Load (GL) is a GI-weighted measure of carbohydrate content, which estimates the impact of carbohydrate intake using the GI while taking into account the amount of carbohydrates that are eaten in a serving. Several studies have demonstrated differences in PPG after consumption of low versus high GI meals, with rapid glucose spikes following high GI meals [9]. Even when comparing foods within the low GI range, foods with lower GI result in lower PPG excursions [10]. Helping people with T1D acheive a diet with a lower glycemic load can improve both their quality of life and their dieabetes-associated complications.

**Fig. 1:**
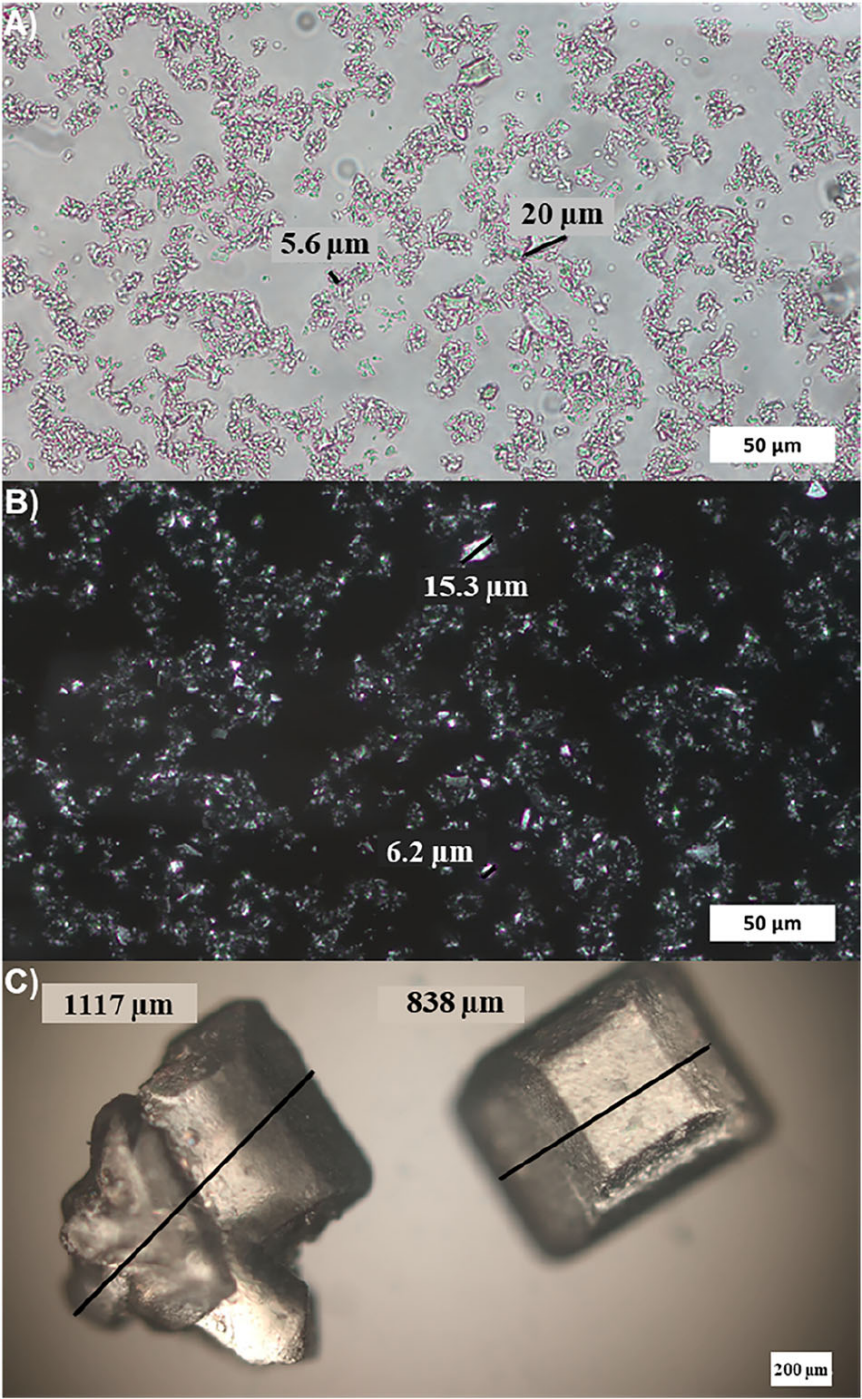
Microscopy images of O’sweet wet milled sugar crystals. O’sweet wet milled sugar crystals under bright fi eld illumination (**A**) and polarized light (**B**), compared to common sugar crystals (**C**). The white “glowing” appearance of the sugar particles under polarized lightin (**B**) proves that the sugar particles have maintained their crystalline structure.

Over recent years, various technologies have been implemented in the attempt to produce sugar-reduced foods that are palatable and do not contain non-nutritive sweeteners. One such innovative technology is milling natural sucrose crystals of 500 µm size to crystalline sugar micro-particles of a median size < 15 µm [11](Fig. 1). When mixed together with oil or fat, this produces a micro-suspension which increases the surface area of the sugar particles, and thus, a lower quantity of sugar is required in order to produce an enhanced perception of sweetness at the taste receptors in the mouth. This provides food products that use up to 80% less sucrose compared to similar, commercially available products, with no reduction in sweetness, no aftertastes of sugar substitutes, and no chemical modifications or additives. The effect of these food products on blood glycaemia has not yet been shown.

In the current study, our primary aim was to compare the glycemic response of people with T1D to 20 grams of the novel sugar-reduced chocolate spread (8% sugar and 1.6 grams of sucrose per serving portion), with the glycemic response to 20 grams of commercially available chocolate spread, (56% sugar and 11 grams sucrose per serving portion). Our secondary aim was to compare the acceptance and palatability of the sucrose-reduced spread with the control spread.

## Materials and Methods

### Study design and participants

This randomized, double-blind, crossover, active-controlled trial evaluated the glycemic response without pre-prandial insulin, and the acceptability of a sucrose-reduced (SR) spread (O’Sweet, Omega 3 Galilee LTD, Teradion Industrial Park, Misgav, Israel) compared to a control spread (Ferrero “Nutella”®, Alba, Piedmont, Italy) in subjects with T1D. The study was conducted at the Institute for Endocrinology and Diabetes, Schneider Children’s Medical Center of Israel.

The study population included young people with T1D, aged 18–35 years, with a diabetes duration of more than 1 year, a BMI between 20–30 kg/m², Hemoglobin A1C < 75 mmol/mol (9%), and who had been using continuous glucose monitors (CGMs). The main exclusion criteria were: (a) An event of acute upper respiratory tract infection within two weeks of enrollment. (b) Gastrointestinal conditions that could impair intestinal absorption or motility (e.g., diabetic gastroparesis, celiac disease, or malabsorption). (c) Chronic illnesses within the past five years (with the exception of well controlled Hashimoto thyroiditis). (d) Use of medications other than insulin (e.g., antibiotics, antifungals, proton pump inhibitors, analgesics) during the study and within two weeks before enrollment. (e) Neuropsychiatric disorders. (f) Known food allergies or intolerances (g) Smoking (h) Self-reported sinus, taste, or smell dysfunction, and (i) Pregnancy or lactation.

### Study procedures

The study comprised two visits conducted within a two-week period. During each visit, participants consumed either 20 g of the SR spread or 20 g of the control spread without administering pre-prandial insulin. The contents of each spread are detailed in Table 1. The randomization was performed by pulling a note from a box with an assignment to one of the two spreads (labeled “A” or “B”). A participant who pulled out a note with the “A” spread in the first visit received the “B” spread on their second visit. As the trial was double-blinded, the company provided the two spreads labeled as “A” or “B” and the identity of the spreads was withheld from the investigators until data analysis.

**Table 1:** Contents of the Study and Control Products.

Contents	Sucrose-Reduced Spread, serving size 20 g	Control Spread, serving size 20 g
**Sucrose%**	8	56
**Fat%**	38.7	31
**Fiber%**	24	~0
**Protein%**	3.7	6.7
**Carbohydrates, g**	5.5	11.5
**Energy, KJ (kcal)**	421.95 (100.8)	451.25 (107.8)
**Glycemic load**	~0	4

The test meals were administered in the morning, after an overnight fast. The glycemic response was measured using both the participants’ personal CGMs and a blood glucose (BG) meter (Abbott’s Freestyle Optium Blood Glucose and Ketone Meter) for two hours following the consumption of the spread. Participants were instructed to arrive at each visit after a night’s fast, unless necessary for hypoglycemia treatment. Subjects using multiple daily insulin injections were instructed to administer long-acting insulin as usual if regularly given in the evening, or directly after the visit if regularly administered in the morning, to avoid a possible peak action of long-acting insulin coinciding with the test. Subjects using insulin pumps were asked to continue their usual basal insulin rate, while participants using an automated insulin system were asked to switch to manual mode before the test meal, to prevent automated administration of extra insulin during the test meal. All participants were instructed to avoid giving bolus insulin (rapid-, ultra-rapid or short-acting) within six hours of the meal visit, unless required to treat hyperglycemia. To ensure that the participants were following instructions, the study physician repeated the instructions over a phone call on the evening before each visit.

The test meals began between 7:00 and 10:00 a.m. The capillary glucose value before the meal had to be > 70 mg/dL and ≤ 180 mg/dL for the test to commence. The participants were instructed to consume the meal within five minutes without administering insulin. Capillary glucose was measured at seven time points using both a BG meter and CGM: 10 min before the meal (-10), at meal start(0), and 15, 30-, 60-, 90-, and 120-minutes post-meal. If glucose levels exceeded 300 mg/dL, plasma ketones (β-hydroxybutyrate) were to be measured. At the end of the visit, participants completed a taste satisfaction questionnaire.

Data extracted from patient files included diabetes duration, and exclusion of co-morbidities. Insulin doses administered in the 3 days prior to each visit were extracted from insulin pump downloads, or in patients using MDI, documented according to patient recall.

### Outcomes

The primary outcome was the difference between pre-meal and peak postprandial glucose levels during each of the two-hour test meals. The secondary outcomes included: 1) The incremental area under the curve (iAUC) using CGM data from meal start to two hours postprandial; 2) iAUC for glucose levels > 180 mg/dL between meal start and two hours postprandial; 3) peak postprandial glucose level during the two-hour postprandial period; 4) time in range (TIR) of 70–180 mg/dL during the postprandial period; 5) percentage of time spent above 180, 250, and 350 mg/dL; and 6) palatability and sweetness acceptability, measured by a taste test questionnaire, based on a Taste Test Guide by Dill & Williams [12]. The taste test questionnaire included six questions. The first five questions assess the participants’ satisfaction with the spread’s appearance, flavor, texture, smell, and overall acceptability. The participants rated each of these parameters on a Likert scale from 1 (“extremely dislike”) to 5 (“extremely like”). The total satisfaction score was calculated as the sum of these five questions. An additional question evaluated the participants’ opinion on the sweetness of the spread, rated on a Likert scale from 1 (“not sweet at all”) to 5 (“too sweet”).

The study was approved by the Rabin Medical Center Institutional Review Board (IRB) and conducted in compliance with the study protocol, the Declaration of Helsinki, and applicable regulatory and Good Clinical Practice guidelines. All participants provided written informed consent before study initiation (Ethical approval code: 0362-23-RMC). The study was registered at ClinicalTrials.gov, ID NCT06334302.

### Statistical analyses

Statistical analyses were performed using SPSS software, version 29 (SPSS, Inc., Chicago, Illinois). Data are presented as mean ± SD for normally distributed variables, median (interquartile range, IQR) for skewed distributions, or number and percentage for categorical variables.

For the comparison of baseline characteristics between participants according to randomization group, we conducted independent samples t-tests or Mann-Whitney U tests for normally and skewed distributed variables, respectively, and chi-square tests or Fisher’s exact test for categorical variables.

For the comparison of glycemic control indices before and during the 2-hour test meal, as well as the taste satisfaction questionnaire scores between the SR and control spread visits, we conducted paired sample analyses: paired sample t-tests or Wilcoxon signed-rank tests for normally and skewed distributed variables, respectively, and McNemar’s test for categorical variables.

Sample size calculation for the primary outcome was based on the following assumptions: a clinically significant difference of 22 mg/dL in plasma glucose, a standard deviation of 40 mg/dL, and a correlation of 0.5 between the two meal conditions [13]. The calculation assumed a significance level of 5% and 80% power. Based on these assumptions, a total of 30 young adults with T1D were recruited for the study.

## Results

The study was conducted between May and September 2024. Thirty young adults, 15 (50%) males with T1D, aged 18–28 years (mean age 23.0 ± 3.0 years), were recruited for the study. The participants met the study’s inclusion criteria, with a mean diabetes duration of 12.4 ± 4.8 years, a mean BMI of 24.4 ± 3.5 kg/m², and a mean hemoglobin A1C of 7.2 ± 0.9%. The mean total daily insulin dose per body weight during the 3 days before each visit was 0.8 ± 0.2 U/kg. Of the participants, 15 (50%) were treated with an insulin pump (Omnipod DASH, Medtronic 640 G or 740 G), 10 (33.3%) were treated with hybrid automated insulin delivery (AID) systems (Medtronic 780 G, Omnipod/Dexcom open-source system) while 5 (16.7%) were treated with multiple daily injections. All participants were using CGMs (Dexcom G6, Medtronic Guardian 4, Flash LIBRE 2, or Eversense). Table 2 presents the participants’ baseline characteristics according to the randomization for the order in which the test meals were administered. All baseline demographic and clinical characteristics were comparable between the two randomization groups.

**Table 2:** Baseline Patient Characteristics According to Initial Randomization.

	SR spread at V1 (*n* = 16)	Control spread at V1 (*n* = 14)	*P*-value
**Sex: Male**	8 (50%)	7 (50%)	1.00
Age (years) Mean ± SD	23.2 ± 2.7	22.7 ± 3.4	0.675
Age at diabetes diagnosis (years) Mean ± SD	10.2 ± 3.9	11.0 ± 3.7	0.554
Duration of diabetes (years) Mean ± SD	13.0 ± 4.2	11.7 ± 5.5	0.470
BMI (kg/m^2^) Mean ± SD	24.1 ± 3.8	24.7 ± 3.4	0.675
HbA1C, mmol/mol (%) Mean ± SD	55 ± 9 (7.2 ± 0.8)	55 ± 12 (7.2 ± 1.1)	0.957
Daily insulin dose (total units) Median (IQR)	46.3 (38.4, 85.5)	49.3 (39.7, 67.1)	0.905
Daily insulin dose per body weight (U/kg) Mean ± SD	0.80 ± 0.25	0.77 ± 0.19	0.736
Daily basal insulin dose Mean ± SD	30.2 ± 14.7	29.7 ± 11.0	0.736

Data are presented as mean ± SD for normally distributed variables, median (interquartile range) for skewed distributions, or number and percentage for categorical variables.

*P*-values are based on independent samples t-tests or Mann-Whitney U tests for normally and skewed distributed variables, respectively, and chi-square tests or Fisher’s exact test for categorical variables.

Daily insulin dose parameters were calculated as the mean of three consecutive days before the study visit.

*SR* sucrose-reduced; *BMI-SDS* body mass index; *MDI* Multiple daily injection.

A comparison of glycemic control indices before and during the 2-hour test meal between the SR and control spread visits, based on CGM and BG meter measurements, is presented in Table 3. The capillary glucose values before the consumption of the spreads (10 min prior, as well as at baseline) were comparable between the two test meals, according to both the CGM and BG meter measurements, and ranged from >70 mg/dL to ≤ 180 mg/dL. Following the consumption of the spreads, the SR spread demonstrated an advantage in all primary and secondary glycemic control outcome parameters compared to the control spread.

**Table 3:** Comparisons of Glycemic Control Indices Before, During and After Consumption of the SR and Control Meal Visits, (*n* = 30).

	SR spread	Control spread	Δ SR minus Control	*P*
**Blood Glucose data**				
Glucose (mg/dL) 10-minutes before meal-test Mean ± SD	114.3 ± 29.6	114.4 ± 37.0	−0.1 ± 36.8	0.988
Glucose (mg/dL) at initiation of meal-test Mean ± SD	113.3 ± 29.6	117.4 ± 38.1	−4.1 ± 36.6	0.545
Peak postprandial glucose (mg/dL) Mean ± SD	157.8 ± 40.9	183.2 ± 47.3	−25.4 ± 45.7	0.005
Δ Pre-meal and peak postprandial glucose (mg/dL) Mean ± SD	44.5 ± 38.2	65.8 ± 35.1	−21.3 ± 33.3	0.002
**CGM data**				
Glucose (mg/dL) 10-minutes before meal-test Mean ± SD	121.7 ± 26.4	124.1 ± 34.5	−2.4 ± 37.6	0.733
Glucose (mg/dL) at initiation of meal-test Mean ± SD	119.9 ± 23.6	123.4 ± 34.4	−3.6 ± 38.1	0.612
Peak postprandial glucose (mg/dL) Mean ± SD	161.5 ± 38.7	190.9 ± 41.5	−33.1 ± 46.7	0.003
Δ Pre-meal and peak postprandial glucose (mg/dL) Mean ± SD	41.7 ± 33.4	67.5 ± 34.2	−25.8 ± 34.9	< 0.001
iAUC (min×mg/dL) Mean ± SD	2638 ± 2910	4909 ± 3506	−2271 ± 3789	0.003
iAUC > 180 mg/dL (min×mg/dl) Median (IQR)	0 (0, 230)	423 (0, 2706)	−270 (−2115, 0)	0.007
TIR 70-180 mg/dL (%) Median (IQR)	100 (61.4, 100)	64.6 (25.0, 100)	14.8 (0, 50.0)	0.030
TAR > 180 mg/dL (%) Median (IQR)	0 (0, 39.6)	35.4 (0, 75.0)	−14.8 (−50.0, 0)	0.030
TAR > 250 mg/dL (%) Median (IQR)	0 (0, 0)	0 (0, 0)	0 (0, 0)	0.102

Data are presented as mean ± SD for normally distributed variables, median (interquartile range) for skewed distributions. *P*-values are based on paired samples analyses: paired samples t-tests or Wilcoxon signed ranks tests for normally and skewed distributed variables, respectively.

*SR* Sucrose-reduced, *CGM* continuous glucose monitoring, *iAUC* Incremental area under the curve, *TIR* Time in range, *TAR* Time above range.

The difference between peak postprandial and pre-meal glucose levels during each of the 2 h test meals (the primary endpoint) was significantly lower following the consumption of the SR spread compared to the control spread (mean 25.8 ± 34.9 mg/dL lower, *P* < 0.001, and 21.3 ± 33.3 mg/dL lower, *P* = 0.002, according to CGM and BG meter measurements, respectively).

All secondary endpoints also showed an advantage for the SR spread. The peak postprandial glucose level during the 2-hour postprandial period was significantly lower following the consumption of the SR spread compared to the control spread (mean 33.1 ± 46.7 mg/dL lower, *P* = 0.003, and 25.4 ± 45.7 mg/dL lower, *P* = 0.005, according to CGM and BG meter measurements, respectively).

**Fig. 2:**
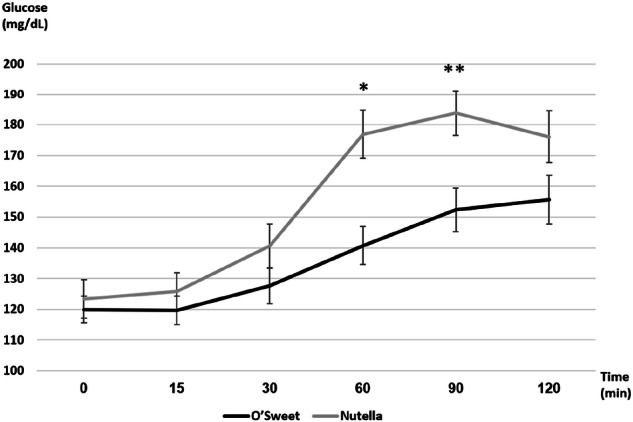
Change in glucose levels (CGM) during 2 hours after consuming 20 grams of the SR spread and 20 grams of the control spread. Data is presented as mean and SE for each of the time points. Paired samples t-test revealed significant differences in glucose levels after 60 and 90 min. *P = 0.002; **P = 0.038.

The iAUC (incremental area under the curve) using CGM data from meal start to two hours postprandial (as presented in Table 3 and Fig. 2) was significantly lower by a mean of 2271 ± 3789 min×mg/dL following the SR spread compared to the control spread (P = 0.003). Paired sample t-tests revealed significantly lower glucose levels at 60 and 90 minutes following the SR spread compared to the control spread. The iAUC for glucose levels > 180 mg/dL between meal start and two hours postprandial (presented in Table 3) was also significantly lower by a median of 270 (IQR -2115, 0) min×mg/dL following the SR spread compared to the control spread (*P* = 0.007).

The median TIR according to CGM measurements during the postprandial period (presented in Table 3) was significantly higher, while the time above range (TAR) > 180 mg/dL was significantly lower, following the SR spread compared to the control spread (TIR 100% and 64.6%, respectively, *P* = 0.030. TAR 0% and 35.4% respectively, *P* = 0.030). None of the participants reached glucose levels ≥ 250 mg/dL following the SR spread, whereas three participants did reach glucose levels ≥ 250 mg/dL but ≤ 300 mg/dL following the control spread.

The taste test questionnaire scores are presented in Table 4. The total satisfaction score was higher for the control spread compared to the SR spread (median score 23 vs. 19 for Nutella and SR, respectively, *P* < 0.001), as were the scores for flavor (median score 4 and 5 respectively, *P* < 0.001), texture (median score 4 and 5 respectively, *P* = 0.004), and acceptability (median score 4 and 5 respectively, *P* < 0.001). However, there was also a significant difference in the sweetness scale rating, favoring the SR spread over control (*P* < 0.001). Twice as many participants rated the SR spread as having the “right degree of sweetness” compared to the control spread (53.3% vs. 26.7%, respectively). None of the participants rated the SR spread as “too sweet,” while 13.3% of the participants rated the control spread as “too sweet.”

**Table 4:** Comparison of Taste Test Questionnaire Scores Between the SR and Control Spread (*n* = 30).

	SR spread	Control spread	*P*-value
**Appearance**	4.0 (3.8, 5.0)	5.0 (4.0, 5.0)	0.084
**Flavor**	4.0 (3.0, 4.0)	5.0 (4.0, 5.0)	< 0.001
**Texture**	4.0 (3.0, 5.0)	5.0 (4.0, 5.0)	0.004
**Smell**	4.0 (3.0, 5.0)	5.0 (4.0, 5.0)	0.254
**Overall acceptability**	4.0 (3.0, 4.0)	5.0 (4.0, 5.0)	< 0.001
**Total satisfaction score**	19 (18, 22)	23 (21, 25)	< 0.001
**Sweet scale n (%)**			
1-Not sweet at all	0	0	
2-Not sweet enough	7 (23.3)	0	
3-Right degree of sweetness	16 (53.3)	8 (26.7)	< 0.001
4-Can be less sweet	7 (23.3)	18 (60.0)	
5-Too sweet	0	4 (13.3)	

Data are presented as median (interquartile range) for skewed distributions, or number and percentage for categorical variables. *P*-values are based on paired-samples Wilcoxon signed ranks tests.

*SR* Sucrose-reduced.

## Discussion

In this randomized controlled crossover trial in people with T1D, the consumption of a novel sugar-reduced product without administration of pre-prandial insulin resulted in significantly reduced PPG excursions compared to the consumption of a similar commercially available product. Overall PPG excursions were lower following consumption of the SR chocolate spread than the control spread, with a smaller pre-meal to peak post-prandial glucose difference, a lower iAUC for postprandial CGM values, a higher TIR, and a lower TAR in the 120 min after the test meal. In light of evidence linking PPG excursions to adverse outcomes, even individuals with well-controlled diabetes may remain at increased risk of developing diabetes-associated complications. Therefore, improved control of PPG excursions should be at the centre of diabetes care.

Our results are in line with previous data, confirming that a lower carbohydrate content produces a lower glycemic response compared to high carbohydrate content both in adults and in children with T1D [14, 15]; that meals with low GI result in lower PPG than meals containing similar carbohydrate content with higher GI [16]; and that a higher content of fiber reduces PPG excursions [5]. Our results also support previous findings regarding benefits of low GL even when comparing foods with relatively low GL [10]. Our results could also have theoretically been influenced by the different amounts of fat and protein in the two spreads (fat% 38.7 and 31 in the SR and control respectively; protein% 3.7 and 6.7% in the SR and control respectively). Both high fat and high protein meal content increase PP hyperglycemias [9]. However, studies show that it takes at least 12.5 grams of protein to affect PPG [17] while both spreads contained a much smaller protein amount. In contrast, the fat content in both spreads was relatively high. The somewhat higher fat% in the SR spread may have delayed the peak glucose level [9] but should have also resulted in higher glucose levels in the SR spread.

The SR product used in this study managed to improve PPG excursion with overall reasonable satisfaction according to taste questionnaires (a median score of 4/5 in each category), albeit scoring less in most categories than the control spread. Of note, the control spread is a commercially available, popular product, while the SR spread is not as widely distributed in Israeli households. Preexisting taste preferences may have been influenced by recurrent exposure to the control spread [18, 19], and this may have affected results. Moreover, as the control spread was produced in Italy, and the SR spread was made in Israel, cultural difference between the populations could explain the differences in sweetness scale rating (perhaps the preference of the Israeli population is for a lower degree of sweetness).

As many as 25% of people with T1D report consuming a higher than recommended sucrose intake [20]. Therefore, there is room for technology to provide better sugar-reduced solutions for this population. Specifically, such products could be beneficial for children with T1D, a population with a tendency for low adherence to diets with restricted CHO and low GI [21, 22]. While technological advances have achieved significant improvement in glycemic control, food choices still play a crucial role in controlling glycemic excursions. Even the use of advanced hybrid AIDs, although significantly improves TIR across the board [23], still does not entirely eliminate PPG excursions, unless coupled with adherence to nutritional recommendations. A recent study conducted in people with T1D using hybrid AIDs, showed that GL, amounts of carbohydrates and of simple sugars consumed, among other factors, were all negative predictors of postprandial TIR (P < 0.05 for all) [24].

Sugar-reduced products are also much needed for the general population. Sugar overconsumption is an independent risk factor for cardiovascular disease as well as many other chronic diseases, including obesity, type 2 diabetes, liver cirrhosis, and dementia—all linked to metabolic perturbations involving dyslipidemia, hypertension, and insulin resistance [25]. World Health Organization guidelines recommend that adults and children reduce their daily intake of free sugars to less than 10% of their total energy intake, with a further reduction to below 5% or roughly 25 grams per day assumed to provide additional health benefits [26]. Unfortunately, a recent review on global sugar intakes found that consumption of free and added sugars overall exceed current dietary guidelines across all population groups, with highest intakes in children and adolescents [27].

The current study presents data regarding the glycemic response to a single food item, and therefore does not resemble a real-life meal but rather provides specific data on the SR product that was tested. Its primary strength lies in its design as a double-blinded, randomized, controlled trial. The crossover design applied facilitates direct comparisons both within and between participants, enhancing the reliability of the findings. Additionally, the use of CGM alongside BG measurements provides comprehensive data on the glycemic response, allowing for precise analysis of postprandial glucose levels. The study is limited by the short duration of postprandial glucose monitoring, with data collected for only 120 min. This restriction, although acceptable per definition [28] may not fully capture the glycemic response dynamics which may be evident over a longer period of time.

## Conclusions

Comparisons of glycemic responses to a novel SR food product compared to a commercially available equivalent showed that an 80% reduction in sucrose and 50% reduction in carbohydrate content resulted in a more favorable glycemic response, without compromising flavor. This new technology could significantly contribute to the ongoing effort to produce sugar-reduced foods for people with diabetes, aimed at improving PPG excursions. Further studies should be conducted to evaluate the impact of similar SR products on glycemia in different populations with diabetes and various meal types.

## Data Availability

The data that support the findings of this study are available from the corresponding author, NFS, upon reasonable request.
